# A functional variant in *HOXA11-AS*, a novel long non-coding RNA, inhibits the oncogenic phenotype of epithelial ovarian cancer

**DOI:** 10.18632/oncotarget.5784

**Published:** 2015-09-22

**Authors:** Edward J. Richards, Jennifer Permuth-Wey, Yajuan Li, Y. Ann Chen, Domenico Coppola, Brett M. Reid, Hui-Yi Lin, Jamie K. Teer, Andrew Berchuck, Michael J. Birrer, Kate Lawrenson, Alvaro N.A. Monteiro, Joellen M. Schildkraut, Ellen L. Goode, Simon A. Gayther, Thomas A. Sellers, Jin Q. Cheng

**Affiliations:** ^1^ Department of Molecular Oncology, Moffitt Cancer Center, Tampa, FL, USA; ^2^ Department of Cancer Epidemiology, Moffitt Cancer Center, Tampa, FL, USA; ^3^ Department of Biostatistics and Bioinformatics, Moffitt Cancer Center, Tampa, FL, USA; ^4^ Department of Anatomic Pathology, Moffitt Cancer Center, Tampa, FL, USA; ^5^ Department of Obstetrics and Gynecology, Duke University Medical Center, Durham, North Carolina, USA; ^6^ Massachusetts General Hospital, Boston, MA, USA; ^7^ Department of Preventive Medicine, Keck School of Medicine, University of Southern California, Norris Comprehensive Cancer Center, Los Angeles, California, USA; ^8^ School of Medicine, Public Health Sciences, University of Virginia, Charlottesville, VA, USA; ^9^ Department of Health Science Research, Division of Epidemiology, Mayo Clinic, Rochester, MN, USA

**Keywords:** ovarian cancer, genetic susceptibility, HOX cluster, long non-coding RNAs, single nucleotide polymorphisms

## Abstract

The homeobox A (*HOXA*) region of protein-coding genes impacts female reproductive system embryogenesis and ovarian carcinogenesis. The 5-prime end of *HOXA* includes three long non-coding RNAs (lncRNAs) *(HOXA10-AS, HOXA11-AS*, and *HOTTIP*) that are underexplored in epithelial ovarian cancer (EOC). We evaluated whether common genetic variants in these lncRNAs are associated with EOC risk and/or have functional roles in EOC development. Using genome-wide association study data from 1,201 serous EOC cases and 2,009 controls, an exonic variant within *HOXA11-AS*, rs17427875 (A>T), was marginally associated with reduced serous EOC risk (OR = 0.88 (95% CI: 0.78-1.01, *p* = 0.06). Functional studies of ectopic expression of *HOXA11-AS* minor allele T in EOC cells showed decreased survival, proliferation, migration, and invasion compared to common allele A expression. Additionally, stable expression of *HOXA11-AS* minor allele T reduced primary tumor growth in mouse xenograft models to a greater extent than common allele A. Furthermore, *HOXA11-AS* expression levels were significantly lower in human EOC tumors than normal ovarian tissues (*p* < 0.05), suggesting that *HOXA11-AS* has a tumor suppressor function in EOC which may be enhanced by the T allele. These findings demonstrate for the first time a role for *HOXA11-AS* in EOC with effects that could be modified by germline variants.

## INTRODUCTION

The homeobox (HOX) family of genes are transcription factors that contribute to embryogenesis and carcinogenesis [1]. They are characterized by highly conserved homeodomains which enable HOX proteins to bind to specific DNA regions and activate or repress transcription of their target genes [2]. In the human genome, *HOX* genes are organized into four clusters (A, B, C, and D) located on four different chromosomes [3]. During the development of the female reproductive system, several ‘*HOXA*’ cluster genes that map to 7p15.2 (*HOXA9, HOXA10, HOXA11,* and *HOXA13*) are expressed uniformly along the Mϋllerian duct axis, though in adults their expression is confined to specific female organs [2, 4]. Since *HOXA* genes promote aberrant epithelial differentiation [4], their expression has been evaluated in epithelial ovarian cancer (EOC), a malignancy that accounts for more deaths in North America than any other cancer of the female reproductive system [5]. Increased expression of several *HOXA* genes has been reported in human EOCs compared to normal ovarian surface epithelial (OSE) precursor tissues [4, 6-9]. The *HOXA* gene cluster is organized into a sense strand containing protein-coding genes and an antisense strand containing non-coding RNA (ncRNA) genes (Supplemental Figure 1A). The 5-prime region of the *HOXA* locus refers to the direction of the sense strand with respect to protein coding genes, with *HOXA13* being the most 5-prime protein-coding gene (Supplemental Figure 1B). The 5-prime region includes three additional protein-coding genes (*HOXA11*, *HOXA10*, and *HOXA9*) and 3 lncRNAs, *HOTTIP, HOXA11-AS,* and *HOXA10-AS*. While several investigations support a role for the *HOXA* cluster of protein-coding genes [2, 4] in ovarian embryogenesis and carcinogenesis, less is known regarding the locally residing lncRNAs and how they may contribute to these processes [10]. Based on recent reports which suggest that lncRNAs in the 5-prime distal region have a functional role in promoting malignant phenotypes [11-13], a rationale exists for their investigation in EOC.

Common germline genetic variants, or single nucleotide polymorphisms (SNPs), affecting lncRNAs have been shown to contribute to the development of multiple cancer types [14-19]. The objective of this investigation was to comprehensively examine inherited genetic variation in the three lncRNAs in the 5′ end of the *HOXA* cluster region (*HOXA10-AS, HOXA11-AS,* and *HOTTIP*) in EOC. In particular, we tested the hypotheses that these variants associated with EOC risk, *in vitro* cell survival or proliferation, *in vitro* migration or invasion, EOC growth *in vivo*, and tumor expression. To examine risk, we evaluated genotype and clinical data from 1,201 invasive serous EOC cases and 2,009 controls from a North American genome-wide association study (GWAS) of EOC (Table 1). To evaluate functional aspects, we tested the most promising candidate SNP for functional impact using *in vitro* and *in vivo* assays. Our results suggest that although germline variants in lncRNA sequences within the *HOXA* cluster are not convincingly associated with EOC risk, these variants could be functional in driving malignant phenotypes associated with cancer. This line of research provides a new opportunity to advance our understanding of *HOXA* cluster-mediated regulation of EOC development.

**Table 1:** Characteristics of participating genome-wide association studies of epithelial ovarian cancer

Study Name	Study Population	Genotyping Platform	Study Type	Number of subjects^1^		
				cases	serous	controls
**North America**						
Mayo Clinic Ovarian Cancer Study	Upper Midwest, USA	Illumina 610K	Clinic based	359	237	520
North Carolina Ovarian Cancer Study	North Carolina, USA	Illumina 610K	Population based	494	285	654
Tampa Bay Ovarian Cancer Study	Tampa, USA	Illumina 610K	Population based	227	146	169
Familial Ovarian Tumor Study	Ontario, Canada	Illumina 610K	Population based	734	401	524
New England Case-Control Study of Ovarian Cancer	New England, USA	Illumina 317K, 370K	Population based	133^2^	132	142
**Total**				**1,947**	**1,201**	**2,009**

1 Totals represent the number of non-Hispanic white Europeans passing genotyping quality control criteria and meeting study site-specific inclusion/exclusion criteria with data available on disease status, age at diagnosis/interview, self-reported racial group, and histologic subtype.

2 Cases from NEC that were evaluated as part of this investigation represent postmenopausal advanced papillary serous carcinomas; 26 of these cases were ascertained as part of a hospital-based pre-operative study.

## RESULTS

### Association of lnRNA SNPs in the *HOXA* cluster with EOC risk

**Figure 1:**
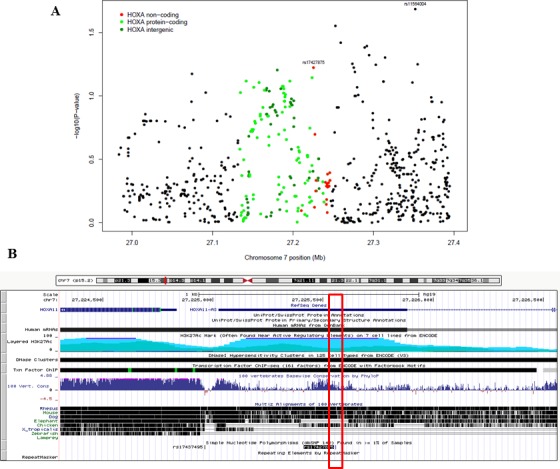
The associations of *HOXA* lncRNA SNP genotypes and surrounding SNPs with epithelial ovarian cancer risk **A.** Regional association plot showing results of association testing between the genotypes of 21 SNPs residing in the 3 *HOXA* lncRNAs (designated by red dots) and the risk of serous EOC (1201 cases, 2009 controls). Also shown are results of association testing for SNPs residing within *HOXA* protein-coding genes (green dots), SNPs falling in intergenic regions (blue dots), and SNPs residing outside the HOXA cluster (black dots). rs17427875, which falls within *HOXA11-AS*, is the top-ranking lncRNA SNP in the region. **B.** Genome browser shot of *HOXA11*, *HOXA11-AS* and the location of the rs17427875, which sits within an evolutionarily conserved region.

We evaluated associations between 21 individual variants in 3 unique lncRNAs in the *HOXA* cluster region and serous EOC susceptibility using data from our GWAS. Figure 1 presents the regional association plot for the SNP-level p-values for the 21 variants, and for reference, 669 variants mapping to the 150 kb flanking regions. No SNPs within the 3 lncRNAs were associated with serous EOC risk at a significance threshold of *p* < 0.05 (Table 2). Only *HOXA11-AS* SNP rs17427875 (A > T; minor allele frequency (MAF) = 0.20) was marginally associated with a reduced risk for serous EOC (OR (95% CI) = 0.88 (0.78-1.01), *p* = 0.060) (Table 2; Figure 1). SNP rs17427875 is not in linkage disequilibrium (r^2^ < 0.01) with rs11564004, the top-ranked SNP in the 150kb region downstream of the *HOXA* cluster (OR (95%CI) = 0.78 (0.63-0.96), *p* = 0.02) (Figure 1A). Per the UCSC Genome Browser, rs17427875 is highly conserved across 100 vertebrates (majority species have only A allele), and is in a conserved peak. It also falls within an H3K27 ChIP region, a DNaseI hypersensitivity cluster, and several transcription factor ChIP regions including EZH2 and POLR2A (Figure 1B).

**Table 2:** Polymorphisms associated with the risk of invasive serous epithelial ovarian cancer

						(1,201 cases; 2,009 controls)				
Gene	Locus	SNP^1^	Al	A2	MAF	OR	LL_CI	UL_CI	P	FDR
HOTTIP	7p15.2	rs3735533^a^	C	T	0.08	0.92	0.76	1.12	0.405	0.078
HOTTIP	7p15.2	rs10233387^b^	G	A	0.45	1.04	0.94	1.15	0.414	0.070
HOTTIP	7p15.2	O7:27245992^a^	G	GC/	0.08	0.93	0.77	1.13	0.463	0.078
HOTTIP	7p15.2	chr7:27245995^a^	G	GC/	0.08	0.93	0.77	1.13	0.463	0.078
HOTTIP	7p16.2	rs3807598^b^	C	G	0.46	1.04	0.94	1.15	0.480	0.070
HOTTIP	7p15.2	c1v7:27244306^a^	TTT	-	0.08	0.93	0.77	1.13	0.486	0.076
HOTTIP	7p15.2	rs1859168^a^	C	A	0.08	0.94	0.77	1.13	0.494	0.070
HOTTIP	7p15.2	rs2023844^a^	A	G	0.08	0.94	0.78	1.14	0.516	0.070
HOTTIP	7p15.2	rs4722675^a^	G	A	0.08	0.94	0.78	1.14	0.516	0.070
HOTTIP	7p15.2	rs929250^a^	T	G	0.08	0.94	0.78	1.14	0.516	0.078
HOTTIP	7p15.2	rs2067087	C	G	0.28	0.96	0.86	1.08	0.516	0.070
HOTTIP	7p15.2	rs2023843^a^	T	C	0.08	0.94	0.78	1.14	0.527	0.070
HOTTIP	7p15.2	chr7:27241878^c^	A	AC1	0.1	1.04	0.88	1.23	0.655	0.070
HOTTIP	7p15.2	rs17501292	T	G	0.18	0.98	0.86	1.11	0.720	0.070
HOTTIP	7p15.2	rs2240042^c^	C	T	0.1	1.02	0.86	1.21	0.832	0.070
HOXA10-AS	7p15.2	chr7:27210428	A	AG	0.44	0.99	0.89	1.09	0.801	0.070
HOXA11-AS	7p15.2	rs17427875	A	T	0.2	0.88	0.78	1.01	0.060	0.070
HOXA11-AS	7p15.2	rs2285724	A	G	0.4	0.94	0.85	1.04	0.201	0.070
HOXA11-AS	7p15.2	chr7:27228665^c^	G	GC/	0.1	1.07	0.90	1.26	0.464	0.070
HOXA11-AS	7p15.2	rs79658629	G	A	0.1	1.05	0.88	1.26	0.563	0.070
HOXA11-AS	7p15.2	rs4722669	C	T	0.08	1.03	0.86	1.24	0.759	0.070

1 Based on human genome build 37: dbSNP 141

Abbreviations: A1 =common allele: A2= minor allele: MAF=minor allele frequency OR=odds ratio, adjusted for study site and the first principal component representing

European ancestry; LL_CI=lower limit of the 95% confidence interval: UL_CI=upper limit of the 95% confidence interval

a, b, c Denote SNP blocks with 2 > 0.8

### *HOXA11-AS* rs17427875 minor allele inhibits cell survival and proliferation more significantly than common allele

**Figure 2:**
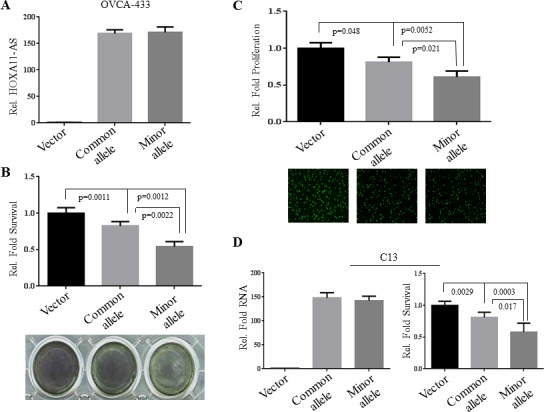
Ectopic expression of the rs17427875 (A > T) variant allele (T) inhibits survival and proliferation **A.** OVCA-433 cells were transfected with vector, common allele, and minor allele of *HOXA11-AS*. Expression of transfected constructs was measured by real-time qPCR. **B.** The transfected cells were subjected to MTT assay for cell viability. **C.** Edu incorporation for DNA synthesis/cell proliferation. **D.** C13 cells were transfected and evaluated for cell survival as described in panels A and B. The survival and growth of vector-transfected cells were normalized to 1.0. Statistical significance was determined using unpaired Student's *t* test.

*HOXA11-AS* is a highly conserved lncRNA across several species (Figure 1B) [20], suggesting that this gene was retained through selective evolutionary pressures. Since the top-ranked candidate lncRNA SNP rs17427875 (A > T) resides within a likely regulatory region within the first exon we tested for allele-specific effects on the cellular phenotypes of *in vitro* models of EOC (Figure 2). We cloned the full-length *HOXA11-AS* common allele construct, and then performed site-directed mutagenesis to generate a plasmid expressing the minor allele. We then transfected OVCA-433 and C13 ovarian cancer cell lines with either the full-length *HOXA11-AS* common allele or the full-length minor allele constructs and assessed phenotypic changes. Forty-eight hours after transfection we observed by real-time PCR that both common and minor allele expression was similar, at ~169 and 171-fold over-expression in OVCA-433, respectively, when compared to vector control (Figure 2A). In both common and minor allele constructs, ectopic expression of the *HOXA11-AS* resulted in a significant reduction of cell survival and proliferation, two major cellular processes associated with EOC development (Figures 2B-2C). Notably, the minor allele resulted in significantly less survival and proliferation compared to the common allele (*p* = 0.0022 and *p* = 0.021). Similar findings were observed in C13 cells (Figure 2D).

### The effects of *HOXA11-AS* rs17427875 on EOC migration and invasion

**Figure 3:**
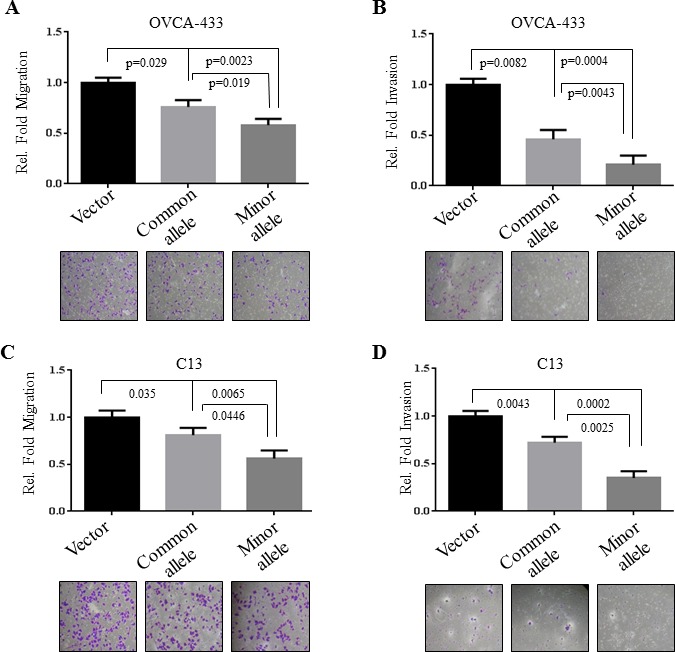
Inhibitory effects of rs17427875 (A > T) on EOC migration and invasion OVCA-433 and C13 cells were transfected with indicated constructs and then assayed for cell **A.** and **C.** migration and **B.** and **D.** invasion using Boyden chamber assays. Data analysis was performed as described in Figure 2.

Epithelial-mesenchymal transition (EMT), migration, and invasion are important cellular phenotypes that are associated with early EOC development, progression and metastasis [21]. We therefore investigated if *HOXA11-AS* SNP rs17427875 regulates cell migration and invasion. Following transfection of minor allele and common allele constructs of *HOXA11-AS* for 48 hours, OVCA-433 and C13 cells were subjected to a two-chamber assay. In OVCA-433, expression of both common allele and minor allele reduced cell migration (*p* = 0.029 and 0.0023, respectively) and invasion (*p* = 0.0082 and 0.0004, respectively). However, the minor allele exhibited a more significant inhibitory effect in both assays than the common allele (*p* = 0.019 and 0.0043, respectively) (Figure 3A-3B). Similar results were obtained in C13 cells (Figure 3C-3D).

Collectively, these findings suggest that the minor allele functions to inhibit oncogenic phenotypes to a more significant extent than the common allele in EOC cells. This is consistent with the epidemiologic trend towards reduced EOC risk among women carrying the *HOXA11-AS* rs17427875 T allele (OR = 0.88, *p* = 0.060).

### *HOXA11-AS* rs17427875 SNP inhibits EOC growth *in vivo*

**Figure 4:**
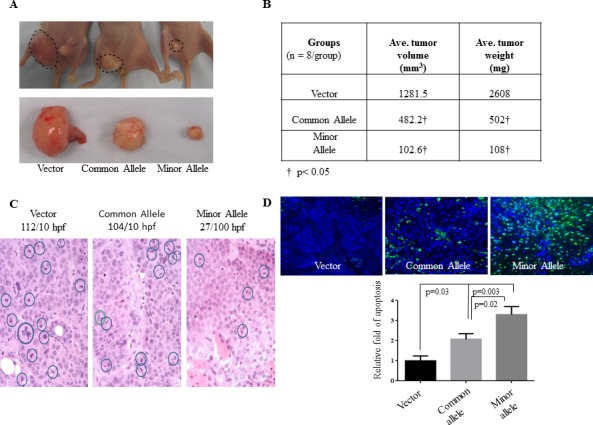
*HOXA11-AS* inhibits EOC tumor growth in xenograft model **A.** C13 cells were stably transfected with vector, common allele, and minor allele constructs. The stable transfected cells were subcutaneously injected to nude mice (4×10^6^ cells/mouse, 8 mice/group) and the image was taken at the 8-week endpoint. **B.** Tumor volume was calculated using standard caliper measurements and tumor weight was measured and quantified. **C.** Histological analysis of H&E staining tumor section. The number of mitotic cells was expressed as mitoses per 10 high-power (x40) microscopic fields. **D.** Tunel assays were performed in xenograft tumor sections. Apoptotic cells were quantified.

Having demonstrated *HOXA11-AS* rs17427875 SNP function in cell culture, we next investigated the effect of this SNP on EOC tumorigenicity in a xenograft model. C13 cells were transfected with vector, common allele, and minor allele constructs. After G418 selection, stably transfected cells were subcutaneously injected into nude mice (4×10^6^/mouse, 8 mice/group). Tumors were engrafted and allowed to grow for 8 weeks at which point the vector control group reached tumor burden endpoint. We observed a reduced tumor size and weight in common allele and minor allele treated mice as compared to the vector control group. Notably, tumor size and weight were significantly less in the minor allele group as compared to the common allele group (Figure 4A and 4B). Furthermore, histological analysis and immuno-staining of tumor sections revealed that expression of the minor allele T reduced mitosis and significantly induced apoptosis when compared to vector control (*p* = 0.003) and common allele (*p* = 0.02, Figure 4C-4D). In contrast, there was no effect of the minor allele on angiogenesis (data not shown). This denotes that the *HOXA11-AS* SNP functions to inhibit tumor growth more significantly than the common allele *in vivo*.

### *HOXA11-AS* expression in EOC

**Figure 5:**
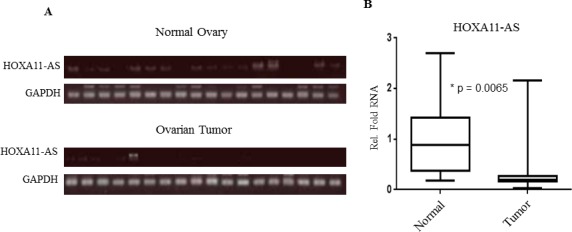
*HOXA11-AS* expression is down-regulated in human EOC tumor **A.** Semi-quantitative RT-PCR analysis of *HOXA11-AS* expression in human EOC tumors and matched normal ovarian tissues. **B.** Quantification and statistical analysis of *HOXA11-AS* expression of EOC and matched normal tissues examined.

In addition, we evaluated *HOXA11-AS* expression and the effect of rs17427875 on *HOXA11-AS* levels in 18 human EOC tumor-normal pairs using semi-quantitative PCR. Using densitometry analysis, expression of *HOXA11-AS* was decreased on average more than 60% in EOC tumor tissue versus normal ovarian tissue (*p* = 6.5×10^−3^; Figure 5A and 5B). The decreased *HOXA11-AS* expression was independent of genotype (Supplemental Figure 2). Taken together, these findings suggest a tumor suppressor role for *HOXA11-AS* in EOC and minor allele T having no effect on *HOXA11-AS* expression.

### Ectopic expression and knockdown of *HOXA11-AS* has no effect on HOXA11 and HOXA13 expression in EOC

**Figure 6:**
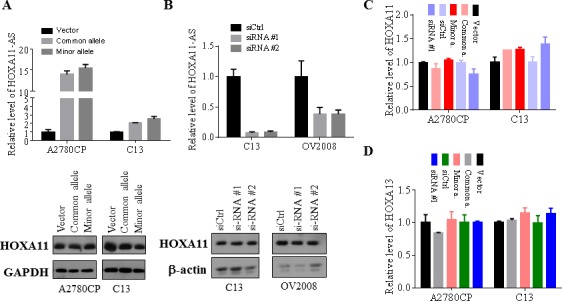
*HOXA11-AS* has no effects on HOXA11 and HOXA13 expression **A.** and **B.** Indicated cell lines were transfected with common, minor allele and siRNAs of HOXA11-AS as well as vector and control siRNA. Following incubation for 48 hours, the cells were subjected to RT-qPCR analysis (upper panels) and western blot with antibodies against HOAX11, GAPDH and β-actin (bottom panels). **C.** and **D.** Indicated cell lines were treated as panels A and B and then were analyzed for HOXA11 and HOXA13 mRNA levels by RT-qPCR.

We also investigated the mechanism by which *HOXA11-AS* inhibits oncogenic phenotypes in EOC. The *HOXA11-AS* is a natural antisense transcript, which is also called antisense lncRNA, located at the 5′ region but not overlapping with the protein coding gene HOXA11 (Supplemental Figure 1). Previous studies have shown that a number of antisense lncRNAs regulate their neighboring genes through a *cis* mechanism [22]. Thus, we assessed HOXA11 protein and mRNA levels following enforced expression and knockdown of *HOXA11-AS* in EOC cell lines. Transfection of common and minor alleles of HOXA11-AS into A2780CP and C13 cells had no effects on HOXA11 expression at both protein (Figure 6A) and mRNA (Figure 6C) levels. Furthermore, expression of HOXA11 was not changed after knockdown of *HOXA11-AS* in C13 and OV2008 as well as A2780CP cells (Figure 6B-6C). Since *HOXA13* is also close to *HOXA11-AS* in an opposite orientation (Supplemental Figure 1), we next examined if *HOXA11-AS* regulates HOXA13. RT-qPCR analysis revealed no significant changes of *HOXA13* mRNA level after expression or knockdown of *HOXA11-AS* (Figure 6D). These findings suggest that *HOXA11-AS* does not regulate *HOXA11* and *HOXA13*.

## DISCUSSION

Dysregulation of the *HOX* cluster of genes has been shown in many human diseases, including EOC [1, 2, 23]. The current study represents the first investigation to focus on the association between genetic variants involving lncRNAs in the *HOXA* gene cluster region and EOC risk and development. Our GWAS identified rs17427875, an exonic variant in *HOXA11-AS* (A > T; MAF = 0.20) as being marginally associated with a decreased risk for serous EOC (OR = 0.88, *P* = 0.06). Regardless of genotype, the *HOXA11-AS* is a tumor-suppressive lncRNA involved in EOC cell survival and proliferation. A series of functional assays provided compelling evidence for an allele-specific effect on tumor growth and several intermediate oncogenic phenotypes: migration, invasion, mitotic index, and apoptosis.

Previous studies have shown that distal *HOXA* protein-coding genes are important in normal ovarian development and that changes in expression occur in ovarian tumors of epithelial origin [2, 4], but the functional role and expression of lncRNAs residing within this region are largely unknown. Through experimental investigation we have shown that ectopic expression of *HOXA11-AS* rs17427875 common and minor allele results in tumor suppressive phenotypes *in vitro*. Notably, expression of minor allele exhibited more significant tumor suppressive activity than common allele. Mouse xenograft studies recapitulate *in vivo* the significant phenotypes associated with expression of the minor allele. These findings are consistent with the observation that rs17427875 was associated with a trend towards decreased risk for serous EOC in the epidemiologic study. Moreover, in 18 RNA normal-tumor pairs from EOC patients, we observed significant down-regulation of *HOXA11-AS* in serous EOC tumors versus normal ovarian tissue. This observation further reinforces our experimental data that *HOXA11-AS* functions as a tumor suppressor.

Antisense lncRNAs can be involved in regulating of the expression of their neighboring genes in *cis* or more distant genes via a *trans* mechanism [22]. Accumulating studies show that a number of antisense lncRNAs demonstrate *cis* regulation of their neighboring genes. For instance, *ANRIL* (antisense non-coding RNA in the *INK4* locus) was shown to silence the *INK4b-ARF-INK4A* locus by recruiting the PRC1 and PRC2 complex [24]. ANRASSF1 (antisense intronic non-coding RASSF1) recruits PRC2 to the *RASSF1A* promoter, which leads to the accumulation of the repressive mark H3K27me3 and reduction in the RASSF1A transcription [25]. However, our data show that knockdown or overexpression of *HOXA11-AS* does not affect expression of its neighboring genes HOXA11 and HOXA13 (Figure 6). Since some lncRNAs have been shown to function as miRNA sponges, we queried the RNA22 database with *HOXA11-AS* sequence and did not find miRNA binding sites within the rs17427875 region (data not shown). In addition, the RNAsnp database analysis shows that minor allele does not cause secondary structure change of *HOXA11-AS* when compared to the common allele (Supplemental Figure 3). These findings suggest that *HOXA11-AS* exerts its cellular function possibly through *trans* regulation of distant genes.

With the rapid growth of identified lncRNAs and disease-associated SNPs, there is a great interest in studying SNPs in lncRNAs [26]. For instance, the rs2839698 TC genotype of lncRNA *H19* was associated with a reduced risk of developing non-muscle-invasive bladder cancer [27]. The SNP rs6983267 within lncRNA *CCAT2* has been implicated in predisposition to colorectal and prostate cancer [28]. A recent study has shown that this SNP affects the expression of *CCAT2*, thereby impacting tumor growth and metastasis in colorectal cancer [29]. Moreover, SNP rs920778, which locates in intron 2 of lncRNA *HOTAIR*, has recently been shown to be associated with esophageal squamous cell carcinoma risk and regulation of *HOTAIR* expression [30]. However, although *HOXA11-AS* was down-regulated in EOC (Figure 5), rs17427875 had no effect on *HOXA11-AS* expression (Supplemental Figure 2). This could potentially be attributed to the small number of evaluated samples, or may indicate a different mechanism of action. It is important to note that while we were performing our *in vitro* and *in vivo* experiments, a larger dataset from the COGS genotyping initiative [31] became available. The association we observed with rs17427875 (A > T) in *HOXA11-AS* was not replicated among an independent set of cases with serous histology (OR (95% CI) = 1.00 (0.95-1.06), *p* = 0.98), and no associations were suggested for other lncRNA SNPs in the region. The basis for lack of consistency in the epidemiologic data is not immediately apparent but may be due to heterogeneity between study populations included in our North American GWAS and those included in COGS, an international genotyping initiative. It is also possible that the initial association observed in the North American GWAS was a false-positive finding. This is a challenge inherent in the detection of weak effects with EOC as an endpoint. Nonetheless, the functional impact of the rs17427875 T minor allele was strong for several oncogenic phenotypes and our data demonstrate that non risk-associated polymorphisms can have allele-specific functionality, which is an important consideration for investigators performing post-GWAS functional studies. Regardless of genotype, this study provides strong evidence for the role of *HOXA11-AS* in EOC.

## MATERIALS AND METHODS

### Study design and population

Details of the study design and population have been previously described [32]. Briefly, we conducted a GWAS of North American EOC case-control studies. Characteristics of the participating studies are summarized in Table 1. Cases were women diagnosed with pathologically-confirmed primary invasive EOC fallopian tube cancer, or primary peritoneal cancer ascertained from clinic-, population-, and hospital-based studies and cancer registries. To increase homogeneity, our primary analysis focused on serous adenocarcinomas, the most common histologic subtype of EOCs [33]. Moreover, most of the cases had high-grade disease. The vast majority of the cases do not have a family history of ovarian or breast cancer in a first-degree relative, and most have not been tested for *BRCA1 or BRCA2* mutations. Controls were women without a current or prior history of EOC with at least one ovary intact at the reference date. All studies had data on disease status, age at diagnosis/interview, self-reported racial group, and histologic subtype. Most studies frequency-matched cases and controls on age group and race. For the present analysis, we focused on subjects of European ancestry since they represent the vast majority of subjects and the burden of EOC is the highest in this racial group [33]. European ancestry was confirmed using principal component analysis [34] with HapMap CEU populations.

### Informed consent

All participants provided written informed consent for their samples and data to be used. The protocol was approved by the institutional review board at each study site.

### Genotyping, quality control, and imputation

Genotypes were generated using different versions of the Illumina Infinium arrays (Table 1). Genotyping methods and sample and SNP quality control procedures have been described previously [35]. Briefly, samples were excluded if they had a call rate < 95%, > 1% discordance, < 80% European ancestry, or ambiguous gender. SNPs were excluded if they had call rates < 95% or if they had minor allele frequencies (MAF) less than 5%. To account for differ­ent marker sets and improve genome coverage, genotype data was imputed for GWAS participants based on data from all 14 populations in the 1000 genomes project (1KGP, version 3, March 2012 release)) as the reference using IMPUTE2 version 2 after pre-phasing with SHAPEIT [36-38]. Before imputation, we excluded poor performing SNPs according to the genotyping success rates, deviation from Hardy Weinberg Equilibrium (HWE) with *P* < 1.0×10^−5^, replicate errors, and rare MAF. To ensure the quality of imputed genotypes, maximum likelihood genotype imputation was carried out and an estimate of the squared correlation between imputed and true genotypes was calculated. Imputed SNPs with an R-square < 0.25 were excluded.

### Identification of variants in candidate HOXA lncRNA cluster genes

The coordinates for each of 3 candidate human *HOXA* lncRNA genes (*HOXA10-AS, HOXA11-AS, and HOTTIP*) were used to identify SNPs falling within the designated gene sequences. To annotate the coordinates for these lncRNAs, we used GENCODEdb (http://www.gencodegenes.org/), the most comprehensive lncRNA annotation available [39]. A total of 72 variants in these 3 genes were represented in the imputed GWAS dataset. After filtering out the variants whose distribution deviated from HWE or had a MAF < 5%, a total of 21 non-coding SNPs falling within the 3 *HOXA* lncRNA genes remained.

### Association testing

We performed SNP-level association tests for the 21 identified SNPs using the pooled GWAS dataset. For each variant, unconditional logistic regression treating alternate alleles as an ordinal variable (a log-additive model) was used to evaluate individual SNP-serous EOC risk associations. Models were adjusted for study site and the first principal component (PC) representing European ancestry. To adjust for multiple comparisons, we estimated the false discovery rate (FDR) and used a corresponding q-value [40] of 10% to declare statistical significance. Statistical analyses were carried out using SAS (Version 9.3; SAS Inc.), PLINK (Version 1.07) [41], Matlab (R2011b; the Mathwork Inc.), and R (Version 3.0.2).

### Quantitative and semi-quantitative RT-PCR

Total RNA was isolated using Trizol reagent following manufacturer's protocol and was used to generate cDNA with High Capacity cDNA Reverse Transcription Kit (Life Technologies, Carlsbad, CA, USA). Quantitative (real-time) PCR was performed with SYBR Green 2x Master Mix (Life Technologies Carlsbad, CA, USA) on ABI HT9600 from Applied Biosystems (Foster City, CA, USA). Cycle threshold (CT) values were generated and analyzed using ABI SDS version 2.3. Delta CTs were normalized to GAPDH reference gene, and ΔΔCT analysis was performed to calculate relative expression of RNA. Patient RNA was extracted from archived human primary EOC tissue and matched adjacent “normal” ovarian surface epithelial cells from each of 18 patients from the Moffitt Cancer Center Total Cancer Care Biorepository [42]. Briefly, 500 ng samples of total RNA were used for RT reaction, and 2ul of the RT product was used for subsequent semi-qPCR reaction. Quantification of PCR was performed using ImageJ software and plotted as a ratio of HOXA11-AS/GAPDH. Real-time PCR primers for HOXA11-AS and GAPDH are listed in Supplementary Table 1. After quantifying mRNA expression levels, we compared the expression levels between matched pairs of samples using the Wilcoxon sign rank test.

**HOXA11-AS common and minor allele expression plasmids** Full-length insert of the common allele of *HOXA11-AS* was amplified using reverse transcription (RT) product as template and cloned to pcDNA3.1 vector (Life Technologies, Carlsbad, CA USA) at EcoRI/XbaI sites. After common allele expression construct was confirmed by sequencing, the minor allele expression plasmid was generated using QuikChange^®^ Site-Directed Mutagenesis Kit (Agilent Technologies, Santa Clara, CA USA) and introducing SNP as point mutation in insert. The primers used for HOXA11-AS site directed mutagenesis are listed in Supplementary Table 1.

### Proliferation and survival assays

OVCA-433 and C13 cell lines were generous gifts from Xianghong Wang (University of Hong Kong) and Benjamin Tsang (Ottawa Hospital and Research Institute), respectively. Both cell lines were cultured under normal conditions using RPMI-1640 supplemented with 10% fetal bovine serum. OVCA-433 and C13 cells were seeded into chamber slides and transfected with vector, common, and minor allele constructs and cultured under G418 selection. EdU nucleoside analogue was allowed to incorporate during DNA synthesis for 1 hour. Cells were processed using Click-iT EdU Imaging Kit (Life Technologies) according to manufacturer's protocol. Relative proliferation was quantified by taking mean and standard deviations of 4 non-biased image fields. To assess overall cell survival, 4×10^4^ cells per well were seeded in 12-well plates and transfected with constructs using 4 replicates. After 6 days selection, overall survival was assayed using 0.5mg/ml MTT reagent (Sigma, St. Louis, MO USA), solubilized in DMSO, and read at 590nm.

### Migration and invasion assays

EOC cells were transfected for 48 hours with vector, common allele, or minor allele *HOXA11-AS* constructs. Cells were washed, trypsinized, and seeded into the upper chamber of Boyden Chambers coated with (invasion) and without (migration) Matrigel. All top chambers contained serum free media while lower chambers had RPMI media containing 10% fetal bovine serum. After 16 hours, top chambers were cleared with cotton swab, washed in PBS, and bottom chamber was stained in crystal violet containing 10% methanol. Migration and invasion were quantified by taking mean and standard deviations of 4 non-biased image fields.

### Mouse xenograft model

Animal studies were approved by the Institutional Animal Care and Use Committee at University of South Florida. Six weeks old female Nu/Nu mice were purchased from Charles River Laboratories (Wilmington, MA USA). C13 cells were transfected with vector, common allele, or minor allele constructs and stably selected with G418 for 2 weeks. Cells were washed in PBS, trypsinized, and resuspended in PBS. Eight mice from each group received 4×10^6^ cells/100ul injection subcutaneously. Tumor volume was measured via standard caliper measurements and tumor weight was calculated at endpoint. Tumor sections were histologically analyzed and were examined with Tunel assay.

### Western blot analysis of *HOXA11-AS* effect on its neighboring gene expression

In addition C13, A2780CP and OV2008 cells, which were gifts from Benjamin Tsang (Ottawa Hospital and Research Institute), were transfected with the common and minor allele plasmids and 2 siRNAs of HOXA11-AS. Following incubation for 48 hours, the cells were subjected to western blot analysis with antibody against HOXA11. The siRNA sequences are listed in Supplementary Table 1.

## SUPPLEMENTARY MATERIAL FIGURES


