# Supplementary material for: A functional variant in HOXA11-AS, a novel long non-coding RNA, inhibits the oncogenic phenotype of epithelial ovarian cancer
Source: Oncotarget. 2015 Sep 22;6(33):34745–57. doi: 10.18632/oncotarget.5784 (PMC4741487; doi:10.18632/oncotarget.5784)
Supplement: Supplementary file 1 [file oncotarget-06-34745-s001.pdf]

A functional variant in *HOXA11-AS*, a novel long non-coding RNA, inhibits the oncogenic phenotype of epithelial ovarian cancer

Supplementary Material

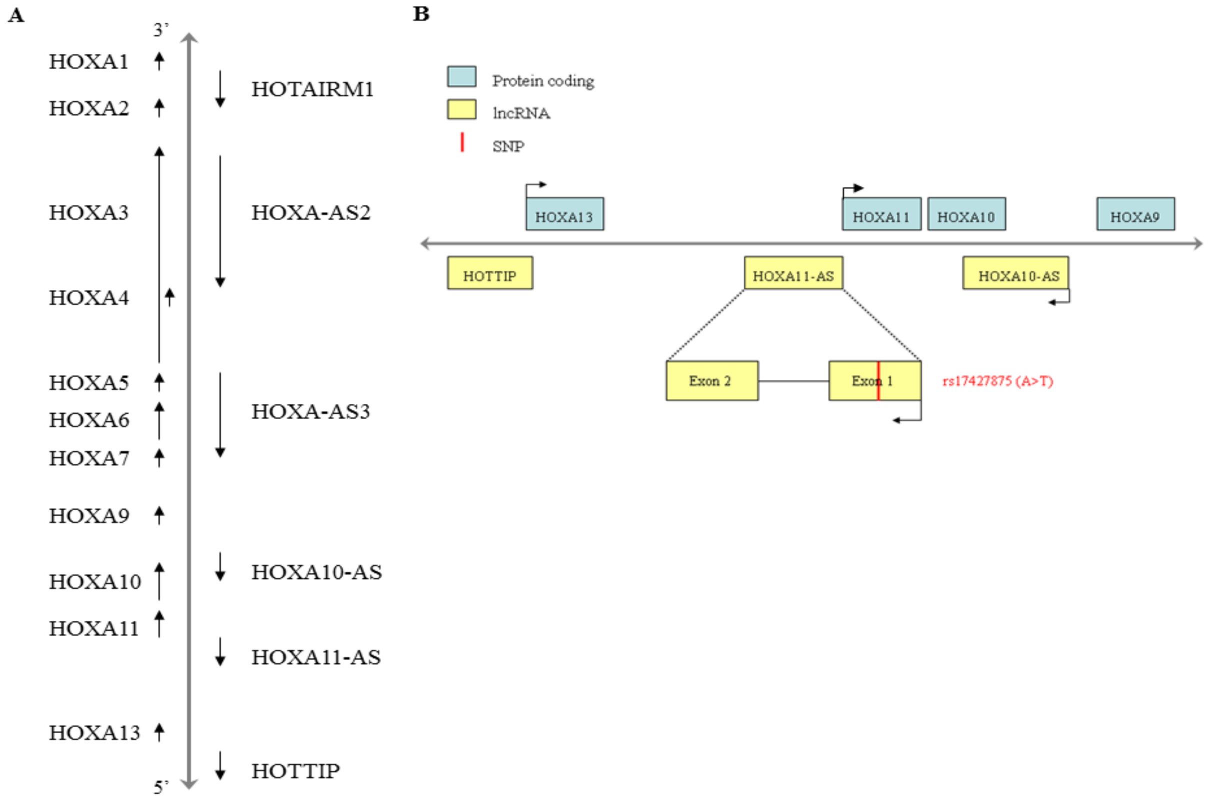

Supplemental Figure 1. *HOXA* Cluster Genomic Landscape (**A**) The *HOXA* gene cluster is comprised of a sense strand of protein coding genes and an antisense strand of lncRNAs. The 5-prime region of protein coding genes locates closest to *HOXA13*, while the 3-prime region corresponds to region closest to *HOXA1*. (**B**) The 5-prime region has reported to be important in ovary development and tumorigenesis. This region contains protein coding genes *HOXA13, HOXA11, HOXA10,* and *HOXA9*, and lncRNAs *HOXA10-AS, HOXA11-AS,* and *HOTTIP*.

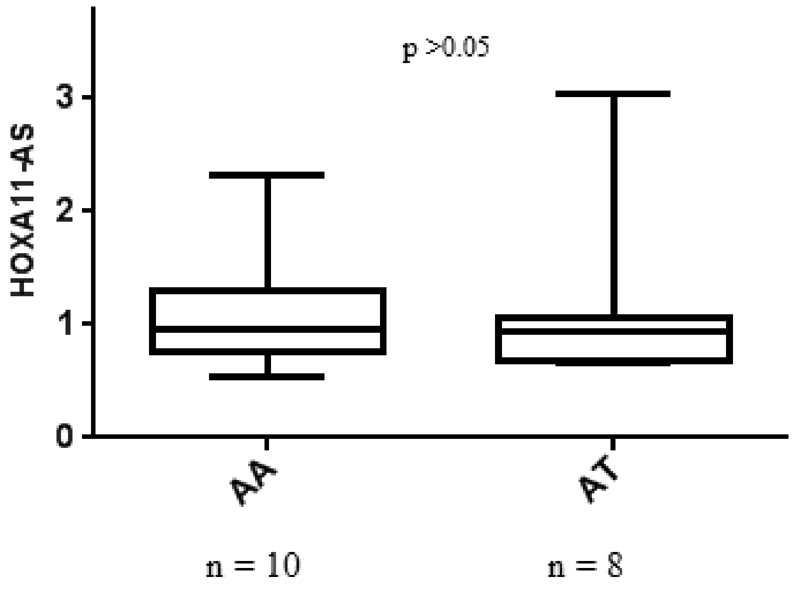

Supplemental Figure 2. ***HOXA11-AS* expression in EOC grouped by rs17427875 A>T genotype.** Real-time PCR analysis of *HOXA11-AS* expression in AA (10 cases) and AT (8 patients) EOC tumor specimens.

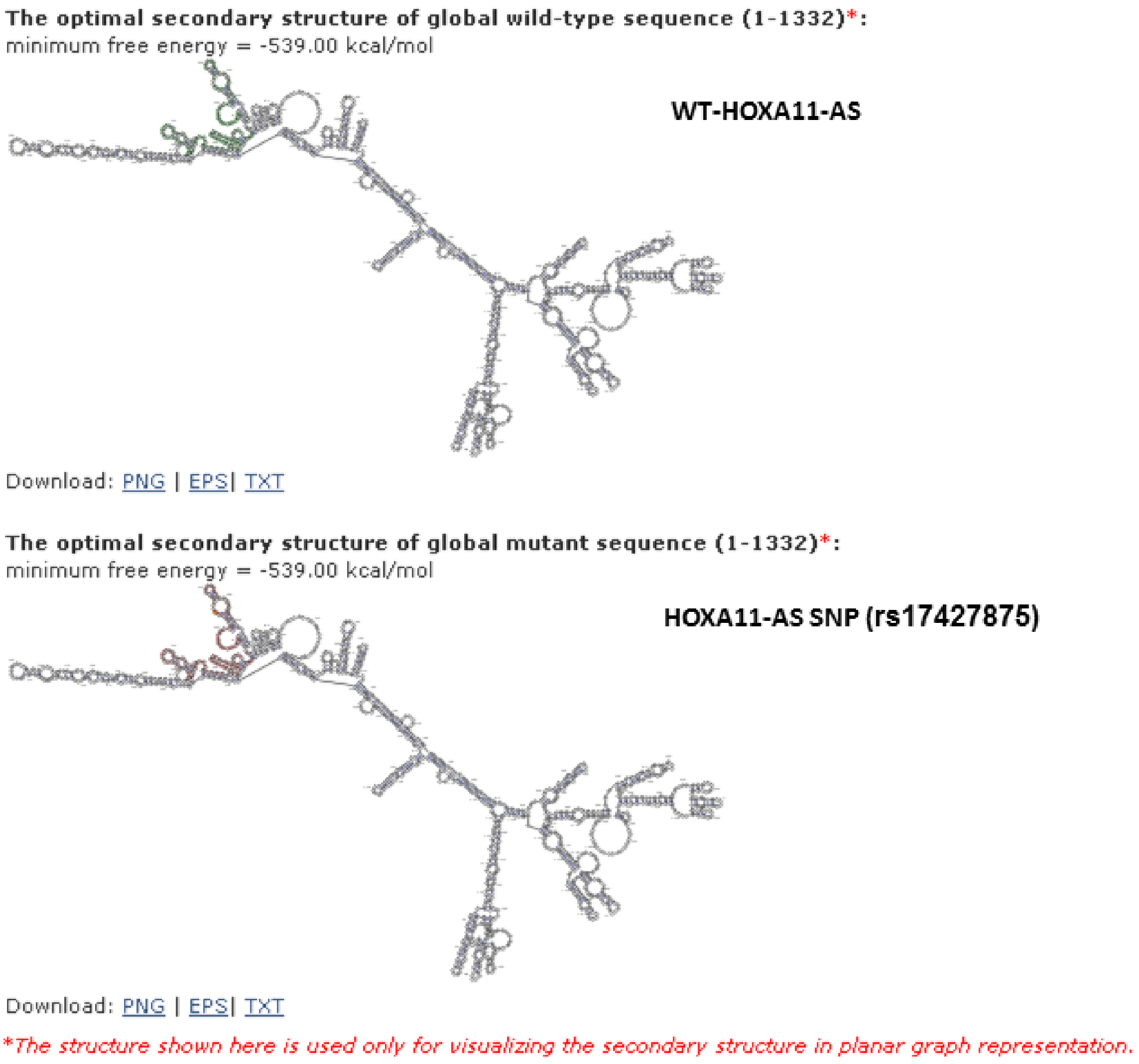

Supplemental Figure 3. Minor allele does not affect the structure of *HOXA11-AS*. RNA structure analysis of common (upper) and minor (bottom) allele of HOXA11-AS using RNAsnp database.
